# Evaluation of a Novel Ambient Light Survey Question in the Cancer Prevention Study-3

**DOI:** 10.3390/ijerph20043658

**Published:** 2023-02-18

**Authors:** W. Ryan Diver, Mariana G. Figueiro, Mark S. Rea, James M. Hodge, W. Dana Flanders, Charlie Zhong, Alpa V. Patel, Susan M. Gapstur

**Affiliations:** 1 Department of Population Science, American Cancer Society, Kennesaw, GA 30144, USA; 2 Light and Health Research Center, Department of Population Health Science and Policy, Icahn School of Medicine at Mount Sinai, New York, NY 10025, USA; 3 Department of Epidemiology, Rollins School of Public Health, Emory University, Atlanta, GA 30322, USA; 4 Winship Cancer Institute, Emory University, Atlanta, GA 30322, USA

**Keywords:** ambient light, survey, validation, illuminance, circadian disruption

## Abstract

Nighttime light exposure may increase cancer risk by disrupting the circadian system. However, there is no well-established survey method for measuring ambient light. In the Cancer Prevention Study-3, 732 men and women answered a light survey based on seven environments. The light environment in the past year was assessed twice, one year apart, and four one-week diaries were collected between the annual surveys. A total of 170 participants wore a meter to measure photopic illuminance and circadian stimulus (CS). Illuminance and CS values were estimated for lighting environments from measured values and evaluated with a cross validation approach. The kappas for self-reported light environment comparing the two annual surveys were 0.61 on workdays and 0.49 on non-workdays. Kappas comparing the annual survey to weekly diaries were 0.71 and 0.57 for work and non-workdays, respectively. Agreement was highest for reporting of darkness (95.3%), non-residential light (86.5%), and household light (75.6%) on workdays. Measured illuminance and CS identified three peaks of light (darkness, indoor lighting, and outdoor daytime light). Estimated illuminance and CS were correlated with the measured values overall (r = 0.77 and r = 0.67, respectively) but were less correlated within each light environment (r = 0.23–0.43). The survey has good validity to assess ambient light for studies of human health.

## 1. Introduction

There is growing evidence that greater nighttime light exposure is associated with higher risk of obesity, diabetes, depression, and cancer [1,2]. For cancer specifically, nighttime light exposure may increase risk via circadian disruption resulting from melatonin suppression, and other biologic responses [3,4,5,6,7]. In human studies, the evidence of an association between nighttime light exposure and risk of some types of cancer primarily result from studies of night shift work, sleep patterns, and circadian-related genes [8,9,10,11]. Night shift work is classified as a probable human carcinogen by the International Agency for Research on Cancer (IARC) [12]. However, nighttime light exposure among night shift workers may not represent the general population. To advance the understanding of light exposure on health outcomes in the general population, methods to reliably and validly assess exposure are needed.

In the Nurses’ Health Study, the Harvard Light Exposure Assessment (H-LEA) [13] questionnaire measured ambient light exposure over a 24 h period. Nurses reported the type of light (ex. halogen lamp, fluorescent lamp, incandescent light, other artificial light sources, natural light and darkness) they were exposed to hourly over 24 h for seven consecutive days. Based on an assumption that the light source type was indicative of light level, illuminance values (lux) were assigned to each type of light. These values were compared to illuminance measured from a light meter worn at eye level by participants. The Spearman correlation between self-reported and measured illuminance was 0.72. 

While results from that study suggest ambient light can be measured using a survey, there are several areas which were not addressed. Importantly, the H-LEA is reliant on a person’s knowledge of the types of lightbulbs in their environment. Participants may not be knowledgeable about the lighting environment, which may consist of a large mix of bulb types, making it difficult to report accurately. Additionally, the H-LEA requires updating as new bulb technologies enter the marketplace, such as light emitting diodes (LED). However, even though bulb technology changes over time, the architectural standards for illuminance [14] in specific types of environments tend to be stable. For example, office and retail workspaces are generally more brightly lit than residential spaces [15]. Therefore, a survey based on the environment would be less affected by changes in technology, allow for a mix of lightbulb types, and not require knowledge of lightbulbs. 

We developed a self-reported light exposure survey based on the lighting environment. In a diverse sample of men and women nationwide, we assessed the 1-year reproducibility and comparative validity of the survey to capture a typical 24 h lighting environment by comparing responses between two surveys completed one year apart and by comparing the information collected on the second survey to four 7-day diaries collected over the course of the previous year. We also compared diary data to measured data from a personal light exposure meter worn by participants for 7 days twice in the year, to assign and validate photopic illuminance and circadian stimulus (CS) values at the reported locations. 

## 2. Materials and Methods

### 2.1. Study Population

Participants were drawn from the American Cancer Society’s Cancer Prevention Study-3 (CPS-3), a prospective cohort of ~304,000 men and women from 35 U.S. states and Puerto Rico [16]. Participants provided a blood sample, had waist circumference measured, and completed a survey on medical, demographic, and lifestyle characteristics at enrollment in 2006–2013. In 2015, a gender and race/ethnicity stratified random sample of CPS-3 participants were invited to enroll in the CPS-3 Activity Validation Sub-study (CPS-3 AVSS). Among the 10,000 participants invited, 1801 pre-registered and consented to participate. The first 300 white women, 150 white men, 150 Latino/as, and 150 African American participants who completed the 2015 CPS-3 follow-up survey were initially enrolled. One selected participant was late to enroll and was replaced, but later completed enrollment, resulting in an extra participant. In total, 751 participants were enrolled in the CPS-3 AVSS.

### 2.2. Ambient Light Survey Questions

The categories of light environment were based on theoretically meaningful differences [15]. Residential lighting standards suggest more illuminance in the kitchen than other spaces of a home. Non-residential working spaces have illuminance standards 2–3 times greater than residences (e.g., offices, retail spaces), except for spaces that are more like residences, such as restaurants and hotels. Outdoor illuminance at mid-day on a cloudless day is orders of magnitude higher than interior lighting; however that light will vary during the day and with the weather. Natural daylight indoors varies highly by the time of day, and the amount of and distance from windows. Therefore, the survey asked about the following seven light environments: darkness (indoors/outdoors), outdoors in daylight (including commuting), indoors with natural light only, household light (excluding kitchen), kitchen light at home, restaurant or hotel light, and other non-residential light (e.g., office, store, gym).

**Figure 1:**
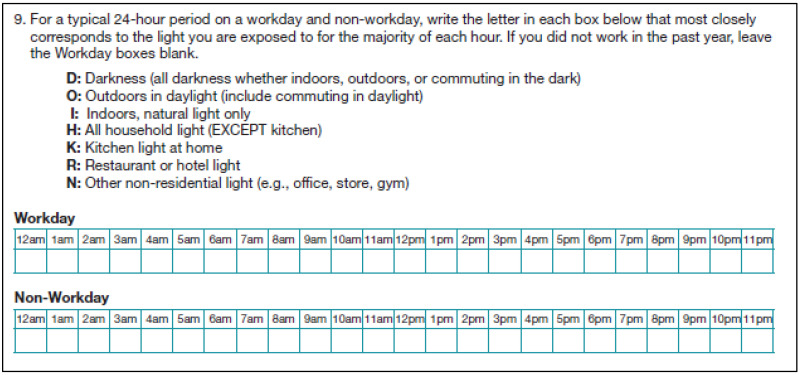
Light environment question included on the pre- and post-validation surveys.

To account for seasonal differences in light exposure and behavior, data was captured over the course of an entire year. In 2015, participants were mailed a pre-validation survey (referred to as the pre-annual survey) and asked to report the typical lighting environment for every hour of the day on a typical workday and non-workday over the past year (Figure 1). After survey return, participants were mailed four 7-day diaries that generally aligned with the following seasons: the summer of 2015 (Q1), fall of 2015 (Q2), winter 2015–2016 (Q3), and spring 2016 (Q4). The 7-day diary included the same questions as those on the survey, but oriented to each of the seven days, and included questions about work hours. Following the last diary, participants were mailed the annual survey again as a post-validation survey (referred to as the post-annual survey). 

Participants were excluded who were missing all light questions on the pre- and/or post-surveys (n = 19), leaving a total of 732 participants in the survey-based analyses.

### 2.3. Measurement of Ambient Light

To measure light exposures during two of the 7-day periods when the diaries were completed, a subset of participants were asked to wear a Daysimeter [17,18]. The Daysimeter was developed by the Lighting Research Center, Rensselaer Polytechnic Institute, to be a photometric measuring system that characterizes retinal optical radiation for the human circadian system and includes an accelerometer. The Daysimeter records light and activity continuously over many days. Through post-processing, the data from the device can be used to determine circadian entrainment and disruption by comparing circadian light/dark patterns with activity/rest patterns [19]. Calibrated photocurrent readings from each of the red, green, blue (RGB) solid-state photosensors can be transformed into measurements of photopic illuminance and circadian light (CL_A_) [20]. Briefly, illuminance is irradiance weighted by the photopic luminous efficiency function (V(λ)), an orthodox measure of the spectral sensitivity of the human fovea, peaking at 555 nm. CL_A_ is irradiance weighted by the spectral sensitivity of the retinal phototransduction mechanisms stimulating the suprachiasmatic nuclei, based on nocturnal melatonin suppression [20]. It is worth noting that some researchers have suggested that the action spectrum of melanopsin, the active photopigment contained within the intrinsically photosensitive retinal ganglion cells (ipRGCs), can be used as a spectral weighting function for circadian system activation [21]. Whereas ipRGCs are the neural lynchpin connecting the retina to the biological clock, several lines of research have clearly shown that melanopsin alone is not an accurate characterization of the spectral sensitivity of the circadian system [22,23,24,25,26,27]. CS is a transformation of CL_A_ into relative units from 0.1, the threshold for circadian system activation, to 0.7, the response saturation. CS is equivalent to the % of melatonin suppression after 1 h exposure to that light. It should be further noted that CS is the only available mathematical characterization of the operating characteristics of the circadian phototransduction mechanism in the human eye from threshold to response saturation. Therefore, we have chosen to use CS in this study. The Daysimeter was worn on a pendant-type necklace outside of their clothing while the participant was awake to track ambient light. Prior to going to bed, participants switched the Daysimeter to a wristband for sleep tracking.

Among the 751 participants enrolled in the CPS-3 AVSS, 150 (75 men and 75 women) were selected to wear the Daysimeter. Within each gender group, we selected an ethnic distribution of 60% Caucasian, 20% African American, and 20% Hispanic. Half of these participants wore the Daysimeter in summer and winter, the remaining half wore the Daysimeter in fall and spring. However, if participants had unusable data (did not follow protocol, less than 3 days of data, deactivated, or device failure) at a time when we were still able to collect 2 quarters of data, then additional participants were selected to maximize the number with 2 quarters of Daysimeter data. In total, 190 men and women were mailed a Daysimeter. Among these participants, 170 had usable data for at least one quarter and were included in analyses. 

### 2.4. Statistical Analysis

Sociodemographic characteristics for the men and women included in this analysis were assessed using distributions of categorical variables and means with standard deviations for continuous data. 

Percent agreement and Cohen’s kappa [28] were used to assess the concordance of responses between the pre-survey and post-survey. Responses were compared for each hour of the day, for each light environment, and for all 24 h combined. Analyses were run overall and separately for workdays and non-workdays. 

To assess the validity of the annual survey in capturing the light environment in a typical work and non-workday during the year, percent agreement and Cohen’s kappa were used to compare the post-annual survey with the information reported in the four 7-day diaries. To compare the single value from the post-annual survey with the 28 days from the diary, the diary data were aggregated to one value for all categorical data by assigning the most commonly reported value on those days. Days were determined to be workdays on the diary if any working hours were reported in the day. Annual agreement was also assessed by gender and race/ethnicity. 

Among participants with Daysimeter data, we provided probability density plots and determined the illuminance and CS values to assign for each light environment reported in the diaries. Linear mixed effect models with random intercepts were fit for illuminance and circadian stimulus with light environments and time of day as predictors, and accounting for within-person correlation. Illuminance data was log transformed to normalize the data prior to modeling. The model was used to calculate estimated illuminance and CS values and bootstrapped 95% prediction intervals for each light environment at each time of day. To validate this modeling approach, we conducted a 10-fold cross validation [29]. Briefly, participants were randomly divided into 10 groups, illuminance and CS values for one group were predicted using a model fit with data from the other 9 groups. This process was repeated for each of the 10 groups and the measured values for each participant were compared to the predicted values. The median absolute error (due to outliers) was calculated to evaluate the performance of this modeling method. Additionally, the predicted values were compared to the observed values using correlation coefficients accounting for repeated measures [30]. Briefly, a modified ANCOVA was fit with the observed value as the outcome and the predicted value as a covariate while treating the participant as a factor. The resulting sum of squares data was used to calculate the repeated measures correlation. Values were assigned using two approaches: assignment of one individual value to each light environment, which assumes the light is the same at all hours of the day, and by assigning unique values for each hour the lighting environment was reported. For hourly estimates, some hours were combined due to sparse data. Sampling distributions of estimated values were provided using bootstrapping [31].

Evaluation of statistics was based on a kappa of 0.6 indicating moderate agreement [28], and a correlation coefficient of ≥0.75 indicating good and 0.5–0.74 indicating moderate correlation, respectively [32]. The survey-based analyses were powered to detect a minimum kappa of 0.66 that is significantly greater the threshold of 0.60. The Daysimeter-based analyses were minimally powered to detect correlation coefficients of 0.84 and 0.66 significantly greater than thresholds of 0.75 and 0.5, respectively.

## 3. Results

There were 296 men and 436 women included in the analysis, with most participants between the ages of 40 and 60 years (Table 1). Most participants were married, highly educated, and employed (18% were not currently working and 12% were working multiple jobs during the year of the survey). Only 8.4% of men and 4.4% of women reported night shift work. There was representation of participants from all regions of the United States. Most participants had complete diary data (mean of 6.96 days/quarter and 23 h/day). Similarly, most Daysimeter participants had data for all 14 days (mean wear time = 20.0 h/day).

**Figure 2:**
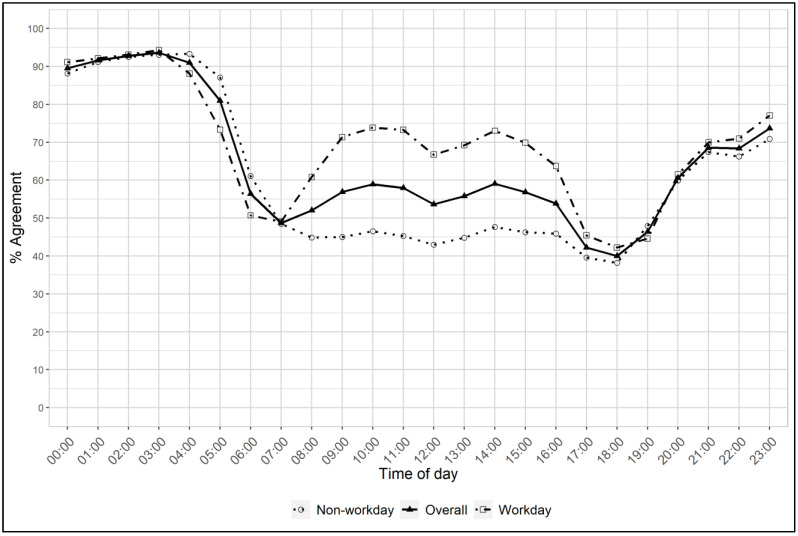
Percent agreement between the pre- and post-annual surveys by time of day for all days (triangles with solid lines), workdays (squares with dash-dot lines), and non-workdays (circles with dotted lines).

The percent agreement and kappa statistics comparing the 1-year reproducibility of the annual survey are shown in Table 2 and Figure 2. Agreement was near moderate for overall light environment (kappa = 0.56), and was higher on workdays than non-workdays. The highest agreement among light environments was “darkness” followed by “other non-residential lighting” on workdays. However, agreement for non-residential lighting was much lower on non-workdays. Restaurant/hotel and kitchen lighting also showed lower agreement. Agreement patterns were similar by gender and race/ethnicity, with slightly poorer agreement in African Americans (Supplementary Table S1). The highest agreement by hour of the day occurred during the nighttime hours, peaking at 94%, while the lowest agreement was at 18:00 (38%). Compared to non-workdays, the agreement was higher for workdays during the typical daytime working hours from 09:00 to 15:00.

The percent agreement and kappa statistics comparing the four weekly diaries with the post-annual survey covering the same period are shown in Table 2. The overall kappas and percent agreement for both workdays and non-workdays were higher than those comparing the two surveys taken 1 year apart. Similarly, agreement was generally higher in all light environments, with the notable exceptions of kitchen lighting and restaurant/hotel lighting on non-workdays. The restaurant/hotel percent agreement for non-workdays was poor (8.9%). When comparing the agreement of the pre-survey with the diaries in the next year, results were similar to the pre- vs. post-annual survey results (Supplementary Table S2). The hourly agreement patterns between the diaries and the post-annual survey were nearly identical to the pattern between the pre- and post-annual surveys completed 1 year apart, except the percent agreement was ~5% higher (Supplementary Figure S1). The highest agreement was 98% at 03:00 and the lowest agreement was 46% at 18:00. 

**Figure 3:**
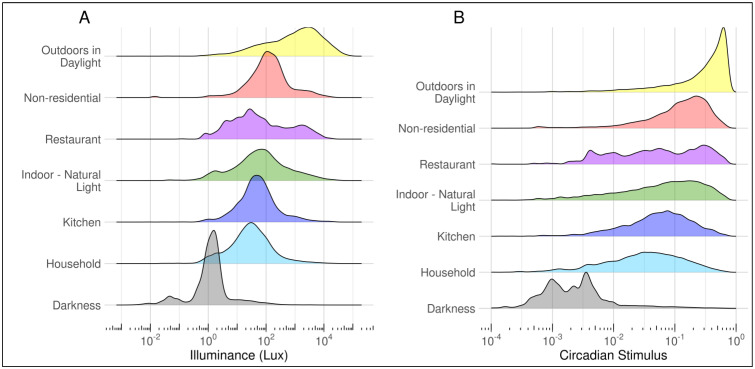
Density plots of measured illuminance (**A**) and circadian stimulus (**B**) by self-reported light environment. The y-axis represents the percent of data within each light environment.

Density plots of measured illuminance and circadian stimulus by lighting environment as reported in the diaries are shown in Figure 3. There were three distinct peaks related to darkness, indoor light, and outdoor light. For illuminance, the bases of the peaks were wide, particularly for outdoor light, indicating large variability in each environment. Similar patterns were shown for CS; however a large portion of outdoor light clustered at the maximum CS value of 0.7. Although the indoor light measurements overlap, there are differences by environment, as shown in Table 3. Other non-residential lighting mostly consists of workplace lighting and is 2–4 times the illuminance of other indoor light environments. Household light had the lowest estimated indoor value. The corresponding CS showed similar patterns, with values ranging from 0.06–0.17 for indoor lighting and 0.39 for outdoor light.

Estimated illuminance and CS values for each light environment were assigned using two methods: one single overall value for each light environment and by hour of day in each environment (Supplementary Table S3). The median error only modestly changed when using hourly values, although there were reductions in the 25th percentile of illuminance for environments with natural light. The overall correlation coefficient using a single value in each light environment was good for illuminance (r = 0.77) and moderate for CS (r = 0.67). Values were slightly improved when using estimates specific to each hour (r = 0.81 and 0.70, respectively). However, the correlations within each light environment when assigning hourly values was moderate to poor, ranging from 0.23 to 0.43. 

## 4. Discussion

We developed and assessed the reproducibility and validity of an ambient light environment survey for use in large epidemiologic studies. Overall, there was moderate agreement in self-reported ambient light exposure over a 24-h period between two surveys completed one year apart, which was higher on workdays than on non-workdays. For specific light environments, there was moderate agreement, with the best agreement for darkness and non-residential lighting. When comparing self-reported light environments from the surveys to the weekly diaries, agreement statistics for both workday and non-workday generally ranged from moderate to good. Measured illuminance showed wide variability within each of the reported illuminated environments. 

Assignment of values to each light environment had good correlation with measured light data overall. This ambient light environment survey adequately captures light exposure at a level useful for epidemiologic studies.

Previous research has generally assessed the ability of a survey to accurately assess light exposure over a short time-period. The H-LEA study evaluated validity over seven days [13], while other studies of ultraviolet radiation (UVR) generally evaluated their instrument in short windows, ranging from 4 to 17 days [33,34]. In this study, we assessed typical light exposure over a year. This is important because light exposure changes seasonally and we wanted the survey to be useable at any time of year. By incorporating data from all seasons, the estimates of light exposure generated in this study represent an average of the entire year. The weekly diaries indicated that the annual survey represented typical weeks from the previous year well. The year-to-year agreement was slightly lower than when compared to diaries, but still displayed moderate correlation. There are multiple reasons survey answers could change over time. First, participants may have true changes to their light exposure from one year to the next. Secondly, by participating in the study, participants may be more aware of their true light exposure at the end of the study than at the start. This training effect may have inflated the statistics for the diaries and post-annual survey in the same year. The modestly lower reproducibility observed in African Americans is consistent with other measures in this cohort [35,36]. Although the magnitude of that difference is small, it will need to be accounted for when evaluating racial and ethnic differences in health outcomes related to light exposure. 

Measured illuminance in this study was different than estimated values in the H-LEA for several light environments, particularly outdoor light, which was almost half of the estimated value (2000 lux). Outdoor light estimates had the largest amount of error in this study due to such a wide range of values captured. Nonetheless, we believe the estimate is an improvement in accuracy over the estimated value in the H-LEA study because it is based on measured data. However, the H-LEA did not provide estimates of error, making this unclear. In this study, illuminance in the non-residential lighting environment is substantially higher than being indoors with only natural light. Other light environments followed patterns expected based on design: kitchen light had greater illuminance than other residential, and restaurant/hotel light was similar to residential. Participants in our study were mostly exposed to non-residential light while working, and we assume the illuminance primarily represents office or other workplace-based lighting. The CS was similar for household environments, ranging from 0.03 to 0.07, while non-residential CS was substantially higher at 0.15. These estimates of CS are similar to data in U.S. federal workers after work in winter (mean CS = 0.09), and at the workplace (seasonal means of 0.19–0.26) [37]. The establishment of real-world illuminance and circadian stimulus values for the survey lighting environments provides more accurate estimates than previous theoretical estimates of lighting. 

Notably, the non-residential CS levels in this study are below recommended guidelines [38] for daytime light exposures and in ranges where health effects have been observed [39]. The recent Underwriters Laboratory design guide (UL DG 24480) suggests that typical interior lighting is not bright enough in the day to ensure circadian entrainment for day-active people. Circadian entrainment is the alignment of the body’s circadian rhythm based on external stimuli, such as light. The UL design guide recommends that individuals need CS levels > 0.3 for at least 2 h during the day and dim light (CS < 0.1) at night. Similar recommendations for greater daytime light exposure and lower nighttime light exposures have been published by a group of interested researchers [21]. Observed CS levels from this study are shown in comparison to the guidelines in Table 4. In this study, over 80% of the measured interior light had a CS below 0.3, including time spent in non-residential spaces such as offices primarily occupied during the day. Most participants were only exposed to CS > 0.3 while outdoors in daylight. However, 78% of measured household light had a CS below 0.1, in line with recommended levels for nighttime. Although, the use of self-luminous displays at home can be bright enough to cause circadian disruption, particularly if viewed for extended periods of time [40]. Results from this study provide an informal survey of U.S. light environments and validate concerns about daytime lighting levels.

Although we were able to identify distinct light environments, the ability to report some environments accurately and distinguish them from other environments was limited over a long timeframe. Assessment of restaurant and hotel light showed low agreement, whether evaluating annual repeatability or comparing weekly diaries to the post-annual survey. Unlike other light environments, restaurant and hotel use is not routine. The ability to average time spent in those environments over a year was poor. Additionally, measured restaurant/hotel light had high variability, possibly representing dimmer candle-lit environments or more highly lit fast-food environments. Restaurant/hotel and kitchen lighting made up the smallest portion of the data reflecting only 0.6% and 4.9% of the annual survey data, respectively. The time of day someone is in the kitchen is variable, and the agreement statistics were all less than 40%. When combining household light with kitchen light and other non-residential light with restaurant/hotel light, estimates were largely unchanged (Supplementary Table S4). As a result, in future CPS-3 annual light surveys, these categories will be collapsed with other non-residential and residential light. Restaurant and kitchen light may be worth capturing in a short-term diary setting. Given the high overlap in measured illuminance and CS for all indoor spaces, it could be argued that indoor light should be captured as one environment. However, the remaining environments were reported well, with small but meaningful differences in measured values. Therefore, further reduction in categories would only result in a loss of precision. For long-term exposure, we recommend collecting data from the five lighting environments that were consistently well captured on workdays and non-workdays (darkness, household light, indoors in natural light, non-residential light, and outdoors in daylight) at an hourly level.

We consistently observed higher kappas and agreement for self-reported light environments on workdays than non-workdays when measured a year apart. Workdays are typically routine, while non-workdays are more likely to be variable. Because an individual’s light environment on non-workdays are likely to be inconsistent (i.e., due to location and/or the time of day), over the course of a year there may be more error in self-reported information. Other factors could also play a role in the worse agreement on non-workdays, including changes to the light environment at home or a complete change of address. While work changes, such as a new job, may also have occurred that reduced workday agreement, it is possible changes related to non-workdays were more common. Nonetheless, the overall kappa comparing the diaries to the post-survey on non-workdays was at the threshold of our pre-defined acceptability. Future use of the survey should account for the distribution of a participant’s work vs. non-workdays by adjustment or stratification. 

The effect of changing light by the time of day was observed for both outdoor and indoor environments. The magnitude of change in outdoor light is over 1000 lux, corresponding to estimated CS values from 0.1 to 0.42 in the daytime. This suggests that using an hourly estimate may be important for outdoor light. While similar changes occurred for indoor light, the magnitude was much smaller, indicating a single overall value is acceptable. Importantly, the non-residential light measured during the nighttime hours is quite low, suggesting that it is not representing bright workplace lighting at night. Non-residential illuminance among subjects who reported working in the nighttime hours was more than double the estimate of subjects not working (26.0 vs. 10.5 lux, respectively). Nighttime lighting in non-residential spaces while not working likely represents dimmer spaces (bars and nightclubs). In our study, the nighttime working illuminance was relatively dim compared to some studies [41], and based on only 12 workers. Caution should be used in applying these estimates to a night-working population. Other nighttime light exposures, such as streetlamps or light from devices, are averaged in with the darkness data and additional questions are needed to assess those components. Nonetheless, the hourly illuminance estimates are a unique aspect of this instrument that improves accuracy in the assignment of values.

Correlation coefficients within specific light environments were moderate to poor when assigning hourly values. This is partially explained by the narrower range of measured values within each environment. For example, the interquartile range of household light illuminance is 10.4–74.6 lux, while the range for all light environments would include Darkness with values <1 lux and Outdoors in Daylight at over 5000 lux. Those large distributions in the overall light environment allow for better discrimination than within environments. Additionally, it suggests that accounting for time of day only modestly improves light estimates within the environment, as seen in the limited improvement in overall correlation when hourly estimates are used. Unsurprisingly, environments that are more impacted by sunlight (Outdoors in Daylight, Indoors Natural Light Only, and Household) had the highest correlations when incorporating time of day. This provides additional support for using hourly values for Outdoors in Daylight, where the daily range of illuminance and circadian stimulus is wide.

The ability of our survey to measure ambient light is comparable to other epidemiologic measures that assessed light and other exposures. The overall correlation coefficient comparing diaries with measured data was similar to estimates from the H-LEA and identical to results limited to day workers (r = 0.77) [13]. A recent analysis comparing a UVR dosimeter with diary estimates of time spent outdoors had a correlation of 0.74 that decreased to 0.57 when comparing the dosimeter to a questionnaire covering the prior month [33]. A study by Glanz et al. identified lower correlations between diaries and UVR exposure in parents of children at pools (r = 0.28–0.29) [42]. Beyond light measures, the correlation coefficients perform comparably to other survey-based measures (sedentary time r = 0.56, alcohol r = 0.79–0.80, energy-intake r = 0.27–0.40) collected in related American Cancer Society studies [36,43].

There are many ways this questionnaire can be used in future research on health outcomes. The survey can be used in its categorical form to determine if different light environments play a role in disease. The light environments can be converted into corresponding illuminance and CS using the estimated values, either the mean estimated values or sampled from the provided distributions, to account for variability (Supplementary Tables S3 and S4). For both categorical and continuous data, calculating total hours spent in certain environments or within certain biologically important thresholds of illuminance or CS are possible. Evaluations can be made using exposure at certain critical times of day, the times of highest or lowest stimulus, or evaluating different patterns of exposure and how they vary from workday to non-workday. Studies of sleep and shiftwork with health outcomes will benefit from additional estimates of light exposure. Future research will focus on identifying the key metrics for use in studies of health outcomes, particularly cancer.

This analysis has several strengths. First, the modification to measure lighting environment instead of bulb type makes the survey less reliant on bulb knowledge, and less susceptible to changes in light technology. While light bulbs may evolve over time, the general overall lighting environment is more stable. Second, the survey was evaluated among men and women, in different race/ethnic groups, and included regional, and employment diversity. In addition, the study evaluated typical light exposure over an entire year and accounted for seasonal light variability as compared with short-term measures typically evaluated. These factors make the light estimates more representative of the general US population exposure and useful for assigning lighting exposures in large population-based studies that are unable to measure light directly. Finally, this is the first study to use real-world light measurements to develop the estimated light values for the survey categories which can be used to estimate exposures in future studies. 

There were several limitations to this research. The study was limited in its ability to address differences by night work status. Only 12 participants with measured ambient light data reported rotating or night shift work. The Daysimeter is a device that must be worn throughout the day, which may not be feasible for all participants, and certain environments may not be conducive to wearing a device around the neck. Placement on the chest may also not fully capture light that enters the eyes, and may be an underestimate of light exposure. However, the measured data should provide a representative level of exposure for each participant. We also had no way to identify Daysimeter wrist location at night (e.g., under or over bedding). Volunteer self-selection may have limited the types of environments evaluated. The study estimated light values that could be used throughout the US at any time of year; however estimates could also be derived that allow for racial/ethnic, seasonal, and/or regional variability, which was not addressed in this study. Although estimated illuminance values and CS were estimated using a statistical method that maintains independence, additional validation of light estimates in an independent population would be useful to confirm our findings. Finally, we only examined one measure of circadian system activation, and others could be applied to this questionnaire [21].

## 5. Conclusions

In conclusion, we developed a novel ambient light environment survey and found it reasonably measured light over both short and long time-periods. We recommend collecting five light environments (darkness, household light, indoors in natural light, non-residential light, and outdoors in daylight) at an hourly level for the highest precision. This survey will be useful in studies that evaluate scientific hypotheses related to light exposure when it is not possible to obtain measured values. The low daytime circadian stimulus values observed in this study are in the range where health effects could be observed and warrant further follow-up. Important next steps will involve incorporating this survey of ambient light with other markers of circadian rhythm disruption to evaluate health outcomes including cancer. 

## Figures and Tables

**Table 1:** Characteristics of subjects in the CPS-3 Activity Validation Sub-study and Daysimeter population.

	Annual Surveys and Diaries (N = 732)		Daysimeter (N = 170)	
Characteristic ^1^	Men	Women	Men	Women
N	296	436	86	84
Age, mean (SD)	49.1 (10.1)	48.1 (9.7)	49.0 (10.4)	47.5 (8.8)
Race				
Black	52 (17.6)	88 (20.2)	17 (19.8)	17 (20.2)
Hispanic	43 (14.5)	67 (15.4)	17 (19.8)	15 (17.9)
White	201 (67.9)	281 (64.4)	52 (60.5)	52 (61.9)
Marital Status				
Married	201 (67.9)	264 (60.6)	60 (69.8)	50 (59.5)
Not Married	42 (14.2)	116 (26.6)	14 (16.3)	18 (21.4)
Missing	53 (17.9)	56 (12.8)	12 (14.0)	16 (19.0)
Education				
<College	52 (17.6)	106 (24.3)	19 (22.1)	17 (20.2)
College +	191 (64.5)	275 (63.1)	53 (61.6)	51 (60.7)
Missing	53 (17.9)	55 (12.6)	14 (16.3)	16 (19.0)
Number of Jobs				
0	52 (17.6)	80 (18.3)	22 (25.6)	10 (11.9)
1	213 (72.0)	296 (67.9)	55 (63.9)	58 (69.0)
2	26 (8.8)	53 (12.2)	9 (10.5)	14 (16.7)
3+	5 (1.7)	7 (1.6)	-	-
Shift Type				
Day Shift Only	219 (74.0)	337 (77.3)	59 (68.6)	66 (78.6)
Rotating Shift	15 (5.1)	13 (3.0)	4 (4.7)	4 (4.8)
Night Shift Only	10 (3.4)	6 (1.4)	1 (1.2)	3 (3.6)
Not working	52 (17.6)	80 (18.3)	22 (25.6)	10 (11.9)
Region				
Northeast	59 (19.9)	55 (12.6)	15 (17.4)	14 (16.7)
South	106 (35.8)	152 (34.9)	38 (44.2)	32 (38.1)
Midwest	89 (30.1)	147 (33.7)	22 (25.6)	29 (34.5)
West	42 (14.2)	82 (18.8)	11 (12.8)	9 (10.7)

^1^ All columns include values for N (%) unless otherwise indicated.

**Table 2:** Agreement of self-reported lighting environment on workdays and non-workdays.

	Pre- vs. Post-Annual Surveys ^1^		Diaries vs. Post-Annual Survey ^2^	
	Workdays	Non-Workdays	Workdays	Non-Workdays
Overall Cohen’s Kappa	0.61	0.49	0.71	0.57
	%	%	%	%
Overall Agreement	69.6	60.7	78.3	66.8
Agreement by Light Environment				
Darkness	88.1	86.5	95.3	94.4
Household light (No Kitchen)	58.8	52.6	75.6	67.5
Kitchen light	35.1	31.9	33.8	27.7
Indoors Natural light only	37.3	48.6	45.5	52.9
Restaurant/Hotel light	35.9	33.0	41.9	8.9
Other Non-residential light	76.5	22.7	86.5	33.8
Outdoors in Daylight	49.7	52.9	47.8	47.9

^1^ Comparison of pre- and post-annual surveys administered 1 year apart. ^2^ Comparison of the most common value from 4 weekly diaries with the post-annual survey for the same year.

**Table 3:** Comparison of measured and estimated illuminance and circadian stimulus by self-reported light environment.

	Diary	Median Measured	Estimated	Median Absolute Error (25th–75th%) ^2^		Correlation (95% CI) ^2^	
Light Environment	Hours	Value (25th–75th%)	Value ^1^	Single ^3^	Hourly ^3^	Single ^3^	Hourly ^3^
		**Illuminance (lux)**					
Darkness	12,201	1.3 (0.8–2.0)	1.4	0.7 (0.3–1.0)	0.8 (0.3–1.3)	-	0.35 (0.34–0.36)
Household light (No Kitchen)	9434	28.7 (10.4–74.6)	26.3	19.4 (10.1–51.8)	20.0 (8.8–56.3)	-	0.43 (0.41–0.44)
Kitchen light	1806	48.6 (21.6–110.1)	47.9	32.84 (16.4–65.3)	34.0 (15.3–75.2)	-	0.34 (0.31–0.37)
Indoors Natural light only	6304	62.2 (16.7–223.8)	72.4	80.1 (48.2–131.4)	71.9 (25.2–144.4)	-	0.43 (0.41–0.44)
Restaurant/Hotel light ^4^	417	43.3 (11.1–347.6)	46.8	-	-	-	-
Other Non-residential light	8002	121.1 (51.7–278.4)	147.9	120.5 (67.6–168.3)	104.0 (49.1–167.8)	-	0.31 (0.29–0.32)
Outdoors in Daylight	5694	1205.1 (185.4–4282.2)	955.0	1037.3 (710.6–3208.3)	1048.3 (361.7–3161.5)	-	0.41 (0.39–0.42)
All Environments Combined				37.5 (1.7–152.4)	31.2 (1.9–141.8)	0.77 (0.77–0.77)	0.81 (0.80–0.81)
		**Circadian stimulus**					
Darkness	12,201	0.00 (0.00–0.00)	0.00	0.00 (0.00–0.00)	0.00 (0.00–0.01)	-	0.29 (0.28–0.31)
Household light (No Kitchen)	9434	0.03 (0.01–0.09)	0.06	0.04 (0.02–0.05)	0.04 (0.02–0.07)	-	0.38 (0.37–0.39)
Kitchen lighting	1806	0.06 (0.02–0.12)	0.09	0.06 (0.03–0.08)	0.05 (0.03–0.09)	-	0.28 (0.25–0.31)
Indoors Natural light only	6304	0.07 (0.02–0.19)	0.13	0.11 (0.06–0.14)	0.09 (0.04–0.14)	-	0.31 (0.29–0.32)
Restaurant/Hotel light ^4^	417	0.06 (0.01–0.21)	0.10	-	-	-	-
Other Non-residential light	8002	0.14 (0.07–0.26)	0.17	0.11 (0.06–0.16)	0.10 (0.05–0.15)	-	0.23 (0.21–0.24)
Outdoors in Daylight	5694	0.39 (0.17–0.58)	0.39	0.20 (0.10–0.29)	0.19 (0.09–0.27)	-	0.31 (0.29–0.32)
All Environments Combined				0.04 (0.00–0.13)	0.04 (0.01–0.12)	0.67 (0.67–0.67)	0.70 (0.70–0.71)

^1^ Median of model-predicted values adjusted for time of day and accounting for repeated measures. ^2^ Errors and repeated measures correlation comparing the measured and predicted values using a 10-fold cross validation approach. Assignment of single values will have a correlation of 0 within each light environment. ^3^ Single values assign one value for each light environment regardless of time of day. Hourly values may be different for each hour of the day. ^4^ There were too few subjects reporting restaurant/hotel light to conduct the 10-fold cross validation.

**Table 4:** Estimated circadian stimulus compared to guidelines.

Light Environment		Estimated ^1^ Circadian Stimulus (CS)	Recommended ^2^ Circadian Stimulus (CS)
Darkness		CS < 0.1	CS < 0.1
Indoor Illumination		0.01 ≤ CS ≤ 0.26	Daytime: CS > 0.3 Evening: CS < 0.1
	Household light (No Kitchen)	0.01 ≤ CS ≤ 0.09	
	Kitchen light	0.02 ≤ CS ≤ 0.12	
	Indoors Natural light only	0.02 ≤ CS ≤ 0.19	
	Restaurant/Hotel light	0.01 ≤ CS ≤ 0.21	
	Other Non-residential light	0.07 ≤ CS ≤ 0.26	
Outdoors in Daylight		0.17 ≤ CS ≤ 0.58	CS > 0.3

^1^ 25th–75th percentile of the estimated values for each light environment. ^2^ Underwriters Laboratories Inc., Design Guideline for Promoting Circadian Entrainment with Light for Day-Active People. In Design Guideline 24480, Underwriters Laboratories, Inc.: Northbrook, IL, USA, 2019.

## Data Availability

The data underlying the findings of this study are restricted by the Emory University Institutional Review Board, who approved the consent forms. Data are available from the American Cancer Society by following the ACS Data Access Procedures (https://www.cancer.org/content/dam/cancer-org/research/epidemiology/cancer-prevention-study-data-access-policies.pdf, accessed on 17 February 2023) for researchers who meet the criteria for access to confidential data.

## Supplementary Materials

The following supporting information can be downloaded at: https://www.mdpi.com/article/10.3390/ijerph20043658/s1, Table S1: Self-reported light environment agreement between pre- and post-annual surveys 1 year apart stratified by gender and race/ethnicity; Table S2: Self-reported light environment agreement between the pre-annual survey and diaries from the next year; Table S3: Estimated illuminance (lux) and circadian stimulus (CS) by time of day and location with boot strapped 95% confidence limits for 7 light environments; Table S4: Estimated illuminance (lux) and circadian stimulus (CS) by time of day and location with boot strapped 95% confidence limits for 5 light environments; Figure S1: Percent agreement between the 4 weekly diaries and the post-annual survey in the same year by time of day for all days, workdays, and non-workdays.

## References

[1] Lunn R.M., Blask D.E., Coogan A.N., Figueiro M.G., Gorman M.R., Hall J.E., Hansen J., Nelson R.J., Panda S., Smolensky M.H. (2017). Health consequences of electric lighting practices in the modern world: A report on the National Toxicology Program’s workshop on shift work at night, artificial light at night, and circadian disruption. Sci. Total Environ..

[2] Kecklund G., Axelsson J. (2016). Health consequences of shift work and insufficient sleep. BMJ.

[3] Haus E.L., Smolensky M.H. (2013). Shift work and cancer risk: Potential mechanistic roles of circadian disruption, light at night, and sleep deprivation. Sleep Med. Rev..

[4] Fekry B., Eckel-Mahan K. (2022). The circadian clock and cancer: Links between circadian disruption and disease Pathology. J. Biochem..

[5] Lopes-Júnior L.C., Veronez L.C. (2022). Circadian rhythms disruption in cancer. Biol. Rhythm. Res..

[6] Samuelsson L.B., Bovbjerg D.H., Roecklein K.A., Hall M.H. (2018). Sleep and circadian disruption and incident breast cancer risk: An evidence-based and theoretical review. Neurosci. Biobehav. Rev..

[7] Stevens R.G., Blask D.E., Brainard G.C., Hansen J., Lockley S.W., Provencio I., Rea M.S., Reinlib L. (2007). Meeting report: The role of environmental lighting and circadian disruption in cancer and other diseases. Environ. Health Perspect..

[8] IARC (2007). Working Group on the Evaluation of Carinogenic Risks to Humans Painting, Firefighting, and Shiftwork.

[9] Sigurdardottir L.G., Valdimarsdottir U.A., Fall K., Rider J.R., Lockley S.W., Schernhammer E., Mucci L.A. (2012). Circadian disruption, sleep loss, and prostate cancer risk: A systematic review of epidemiologic studies. Cancer Epidemiol. Biomark. Prev..

[10] Travis R.C., Balkwill A., Fensom G.K., Appleby P.N., Reeves G.K., Wang X.S., Roddam A.W., Gathani T., Peto R., Green J. (2016). Night Shift Work and Breast Cancer Incidence: Three Prospective Studies and Meta-analysis of Published Studies. J. Natl. Cancer Inst..

[11] Wendeu-Foyet M.G., Menegaux F. (2017). Circadian Disruption and Prostate Cancer Risk: An Updated Review of Epidemiological Evidences. Cancer Epidemiol. Biomark. Prev..

[12] IARC (2019). Monographs Vol 124 Group, Carcinogenicity of night shift work. Lancet Oncol..

[13] Bajaj A., Rosner B., Lockley S.W., Schernhammer E.S. (2011). Validation of a light questionnaire with real-life photopic illuminance measurements: The Harvard Light Exposure Assessment questionnaire. Cancer Epidemiol. Biomark. Prev..

[14] Illuminating Engineering Society IES Lighting Library. https://www.ies.org/standards/ies-lighting-library/.

[15] Rea M.S. (2000). IESNA Lighting Handbook: Reference and Application.

[16] Patel A.V., Jacobs E.J., Dudas D.M., Briggs P.J., Lichtman C.J., Bain E.B., Stevens V.L., McCullough M.L., Teras L.R., Campbell P.T. (2017). The American Cancer Society’s Cancer Prevention Study 3 (CPS-3): Recruitment, study design, and baseline characteristics. Cancer.

[17] Figueiro M.G., Hamner R., Bierman A., Rea M.S. (2013). Comparisons of three practical field devices used to measure personal light exposures and activity levels. Light Res. Technol..

[18] Lighting Research Center the Daysimeter. http://www.lrc.rpi.edu/programs/lightHealth/LightandDaysimeter.asp.

[19] Miller D., Bierman A., Figueiro M., Schernhammer E., Rea M. (2010). Ecological measurements of light exposure, activity, and circadian disruption. Light Res. Technol..

[20] Rea M.S., Figueiro M.G., Bierman A., Bullough J.D. (2010). Circadian light. J. Circadian Rhythm..

[21] Brown T.M., Brainard G.C., Cajochen C., Czeisler C.A., Hanifin J.P., Lockley S.W., Lucas R.J., Munch M., O’Hagan J.B., Peirson S.N. (2022). Recommendations for daytime, evening, and nighttime indoor light exposure to best support physiology, sleep, and wakefulness in healthy adults. PLoS Biol..

[22] Hattar S., Lucas R.J., Mrosovsky N., Thompson S., Douglas R.H., Hankins M.W., Lem J., Biel M., Hofmann F., Foster R.G. (2003). Melanopsin and rod-cone photoreceptive systems account for all major accessory visual functions in mice. Nature.

[23] Matynia A., Parikh S., Chen B., Kim P., McNeill D.S., Nusinowitz S., Evans C., Gorin M.B. (2012). Intrinsically photosensitive retinal ganglion cells are the primary but not exclusive circuit for light aversion. Exp. Eye Res..

[24] Mure L.S., Vinberg F., Hanneken A., Panda S. (2019). Functional diversity of human intrinsically photosensitive retinal ganglion cells. Science.

[25] Rea M.S., Nagare R., Figueiro M.G. (2020). Modeling Circadian Phototransduction: Retinal Neurophysiology and Neuroanatomy. Front. Neurosci..

[26] Rea M.S., Nagare R., Figueiro M.G. (2021). Modeling Circadian Phototransduction: Quantitative Predictions of Psychophysical Data. Front. Neurosci..

[27] Ruby N.F., Brennan T.J., Xie X., Cao V., Franken P., Heller H.C., O’Hara B.F. (2002). Role of melanopsin in circadian responses to light. Science.

[28] McHugh M.L. (2012). Interrater reliability: The kappa statistic. Biochem. Med..

[29] Hastie T., Tibshirani R., Friedman J. (2009). The Elements of Statistical Learning: Data Mining, Inference, and Prediction.

[30] Bakdash J.Z., Marusich L.R. (2017). Repeated Measures Correlation. Front. Psychol..

[31] Fu W.J., Carroll R.J., Wang S. (2005). Estimating misclassification error with small samples via bootstrap cross-validation. Bioinformatics.

[32] Koo T.K., Li M.Y. (2016). A Guideline of Selecting and Reporting Intraclass Correlation Coefficients for Reliability Research. J. Chiropr. Med..

[33] Cust A.E., Fenton G.L., Smit A.K., Espinoza D., Dobbinson S., Brodie A., Dang H.T.C., Kimlin M.G. (2018). Validation of Questionnaire and Diary Measures of Time Outdoors Against an Objective Measure of Personal Ultraviolet Radiation Exposure. Photochem. Photobiol..

[34] Thieden E., Agren M.S., Wulf H.C. (2001). Solar UVR exposures of indoor workers in a Working and a Holiday Period assessed by personal dosimeters and sun exposure diaries. Photodermatol. Photoimmunol. Photomed..

[35] Troeschel A.N., Hartman T.J., Flanders W.D., Wang Y., Hodge R.A., McCullough L.E., Mitchell D.C., Sampson L., Patel A.V., McCullough M.L. (2020). The American Cancer Society Cancer Prevention Study-3 FFQ Has Reasonable Validity and Reproducibility for Food Groups and a Diet Quality Score. J. Nutr..

[36] Rees-Punia E., Matthews C.E., Evans E.M., Keadle S.K., Anderson R.L., Gay J.L., Schmidt M.D., Gapstur S.M., Patel A.V. (2019). Demographic-specific Validity of the Cancer Prevention Study-3 Sedentary Time Survey. Med. Sci. Sports Exerc..

[37] Figueiro M.G., Rea M.S. (2016). Office lighting and personal light exposures in two seasons: Impact on sleep and mood. Light. Res. Technol..

[38] Underwriters Laboratories Inc (2019). Design Guideline for Promoting Circadian Entrainment with Light for Day-Active People. Design Guideline 24480.

[39] Figueiro M.G., Steverson B., Heerwagen J., Kampschroer K., Hunter C.M., Gonzales K., Plitnick B., Rea M.S. (2017). The impact of daytime light exposures on sleep and mood in office workers. Sleep Health.

[40] Rea M.S., Nagare R., Figueiro M.G. (2020). Predictions of melatonin suppression during the early biological night and their implications for residential light exposures prior to sleeping. Sci. Rep..

[41] Hunter C.M., Figueiro M.G. (2017). Measuring Light at Night and Melatonin Levels in Shift Workers: A Review of the Literature. Biol. Res. Nurs..

[42] Glanz K., Gies P., O’Riordan D.L., Elliott T., Nehl E., McCarty F., Davis E. (2010). Validity of self-reported solar UVR exposure compared with objectively measured UVR exposure. Cancer Epidemiol. Biomark. Prev..

[43] Flagg E.W., Coates R.J., Calle E.E., Potischman N., Thun M.J. (2000). Validation of the American Cancer Society Cancer Prevention Study II Nutrition Survey Cohort Food Frequency Questionnaire. Epidemiology.

