# Supplementary material for: Evaluation of a Novel Ambient Light Survey Question in the Cancer Prevention Study-3
Source: Int J Environ Res Public Health. 2023 Feb 18;20(4):3658. doi: 10.3390/ijerph20043658 (PMC9959116; doi:10.3390/ijerph20043658)

Supplemental Data for "Evaluation of a Novel Ambient Light Survey Question in the Cancer Prevention Study-3"

Supplementary Table S1. Self-reported light environment agreement between annual surveys 1-year apart stratified by gender and race/ethnicity.

Supplementary Table S2. Self-reported light environment agreement between the pre-annual survey and diaries from the next year.

Supplementary Table S3. Estimated illuminance (lux) and circadian stimulus (CS) by time of day and location with boot strapped 95% confidence limits for 7 light environments.

Supplementary Table S4. Estimated illuminance (lux) and circadian stimulus (CS) by time of day and location with boot strapped 95% confidence limits for 5 light environments.

Supplementary Figure S1. Percent agreement between the 4 weekly-diaries and the annual survey in the same year by time of day for all days (red triangles with solid lines), workdays (blue squares with dash-dot lines), and non-workdays (green circles with dotted lines).

Supplementary Table S1. Self-reported light environment agreement between annual surveys 1-year apart stratified by gender and race/ethnicity.

	**Gender**				**Race/Ethnicity**					
	**Women**		**Men**		**African American**		**Hispanic**		**White**	
	**Workdays**	**Non-Workdays**	**Workdays**	**Non-Workdays**	**Workdays**	**Non-Workdays**	**Workdays**	**Non-Workdays**	**Workdays**	**Non-Workdays**
**Overall Kappa**	0.61	0.49	0.61	0.49	0.54	0.41	0.63	0.51	0.63	0.51
	%	%	%	%	%	%	%	%	%	%
**Overall Agreement**	69.6	60.6	69.4	60.8	63.4	53.9	71	61.6	71	62.4
**Agreement by Light Environment**										
Darkness	88.8	86.8	87.0	86.1	83.3	80.0	90.2	85.9	88.8	88.4
Household light (No Kitchen)	58.3	53.5	59.7	51.4	51.3	44.7	54.3	50.5	62.0	55.4
Kitchen Light	35.5	33.7	34.4	28.2	36.0	23.1	27.2	29.9	37.2	35.6
Indoors Natural light only	38.6	50.0	35.4	46.4	31.9	47.8	50.3	52.4	36.1	47.9
Restaurant/Hotel light	31.4	31.3	44.4	35.4	N/A	6.7	N/A	33.3	38.8	37.8
Other Non-residential light	77.3	26.0	75.5	15.8	68.9	19.1	80.7	23.0	77.8	24.7
Outdoors in Daylight	43.3	48.1	56.1	58.1	52.4	50.5	51.6	59.5	48.3	52.0

Supplementary Table S2. Self-reported light environment agreement between the pre-annual survey and diaries from the next year.

	Pre-Annual Survey vs Diaries [1]	
	**Workdays**	**Non-workdays**
**Overall Cohens Kappa**	0.61	0.50
	%	%
**Overall Agreement**	70.0	61.5
**Agreement by Light Environment**		
Darkness	84.1	83.3
Household light (No Kitchen)	56.1	53.1
Kitchen light	43.5	48.0
Indoors Natural light only	46.3	53.7
Restaurant/Hotel light	73.1	33.3
Other Non-residential light	76.5	14.63
Outdoors in Daylight	51.6	54.3

[1] Comparison of the most common value from 4-weekly diaries with an annual survey representing the previous year.

Supplementary Table S3. Estimated illuminance (lux) and circadian stimulus (CS) by time of day and location with boot strapped 95% confidence limits for 7 light environments.

	Darkness		Household light (No Kitchen)		Kitchen Light		Indoors Natural light only		Restaurant/Hotel light		Other Non-Residential light		Outdoors in Daylight	
**Time[a]**	**lux**	**CS**	**Lux**	**CS**	**lux**	**CS**	**lux**	**CS**	**lux**	**CS**	**lux**	**CS**	**lux**	**CS**
0:00	1.0 (0.9, 1.1)	0.00 (0.00, 0.01)	9.3 (7.7, 11.2)	0.03 (0.02, 0.04)	18.5 (12.7, 26.7)	0.04 (0.01, 0.07)	5.6 (3.9, 7.9)	0.02 (0.00, 0.05)	7.7 (0.6, 102.3)	0.07 (-0.12, 0.26)	6.5 (5.4, 7.9)	0.07 (0.06, 0.09)	3.5 (1.8, 7.0)	0.03 (-0.04, 0.1)
1:00	0.9 (0.8, 1.0)	0.00 (0.00, 0.00)	8.1 (6.5, 10.2)	0.03 (0.02, 0.05)	18.5 (12.7, 26.7)	0.04 (0.01, 0.07)	5.6 (3.9, 7.9)	0.02 (0.00, 0.05)	10.2 (0.8, 126.8)	0.07 (-0.13, 0.25)	6.5 (5.4, 7.9)	0.07 (0.06, 0.09)	3.5 (1.8, 7.0)	0.03 (-0.04, 0.1)
2:00	0.9 (0.8, 1.0)	0.00 (0.00, 0.00)	6.3 (4.8, 8.3)	0.03 (0.01, 0.05)	18.5 (12.7, 26.7)	0.04 (0.01, 0.07)	5.6 (3.9, 7.9)	0.02 (0.00, 0.05)	10.6 (0.8, 108.9)	0.07 (-0.11, 0.27)	6.5 (5.4, 7.9)	0.07 (0.06, 0.09)	3.5 (1.8, 7.0)	0.03 (-0.04, 0.1)
3:00	0.9 (0.8, 1.0)	0.00 (0.00, 0.00)	8.2 (6.2, 10.9)	0.03 (0.02, 0.05)	18.5 (12.7, 26.7)	0.04 (0.01, 0.07)	5.6 (3.9, 7.9)	0.02 (0.00, 0.05)	5.6 (0.4, 69.7)	0.07 (-0.12, 0.24)	6.5 (5.4, 7.9)	0.07 (0.06, 0.09)	3.5 (1.8, 7.0)	0.03 (-0.04, 0.1)
4:00	1.0 (0.9, 1.2)	0.01 (0.00, 0.01)	11.5 (9.1, 14.6)	0.05 (0.04, 0.06)	18.5 (12.7, 26.7)	0.04 (0.01, 0.07)	5.6 (3.9, 7.9)	0.02 (0.00, 0.05)	9.7 (0.7, 104.6)	0.07 (-0.12, 0.26)	6.5 (5.4, 7.9)	0.07 (0.06, 0.09)	3.5 (1.8, 7.0)	0.03 (-0.04, 0.1)
5:00	1.3 (1.2, 1.5)	0.01 (0.01, 0.01)	18.4 (15.7, 21.4)	0.06 (0.05, 0.07)	26.8 (19.5, 36.2)	0.06 (0.04, 0.09)	4.9 (3.4, 7.0)	0.03 (0.00, 0.05)	7.5 (0.9, 56.9)	0.06 (-0.08, 0.21)	17.6 (12.7, 23.1)	0.09 (0.07, 0.12)	35.1 (16.6, 70.0)	0.10 (0.02, 0.18)
6:00	2.1 (1.8, 2.4)	0.02 (0.02, 0.02)	34.1 (29.9, 39.3)	0.08 (0.08, 0.09)	39.0 (32.1, 47.4)	0.09 (0.08, 0.1)	7.0 (5.7, 8.6)	0.04 (0.03, 0.06)	113.7 (23.7, 531.1)	0.14 (0.02, 0.25)	67.4 (51.5, 84.6)	0.13 (0.11, 0.15)	92.5 (68.7, 122.4)	0.20 (0.17, 0.23)
7:00	3.8 (3.2, 4.6)	0.03 (0.03, 0.04)	45.4 (40.0, 52.0)	0.10 (0.09, 0.11)	60.1 (49.9, 74.1)	0.11 (0.1, 0.12)	15.2 (12.7, 18.2)	0.07 (0.06, 0.09)	37.1 (12.4, 113.4)	0.12 (0.04, 0.2)	125.6 (105.2, 147.7)	0.17 (0.16, 0.18)	307.5 (254.0, 370.2)	0.3 (0.28, 0.32)
8:00	4.4 (3.4, 5.7)	0.06 (0.05, 0.07)	74.8 (64.9, 87.2)	0.12 (0.12, 0.13)	86.1 (68.1, 110.0)	0.12 (0.11, 0.14)	44.8 (37.4, 53.1)	0.12 (0.10, 0.13)	119.9 (55.4, 262.0)	0.15 (0.09, 0.21)	151.8 (132.4, 174.1)	0.19 (0.18, 0.20)	713.5 (596.3, 848.3)	0.36 (0.34, 0.38)
9:00	2.6 (1.8, 3.6)	0.02 (0.01, 0.03)	84.3 (71.9, 98.7)	0.13 (0.12, 0.14)	114.6 (82.9, 159.4)	0.14 (0.12, 0.16)	88.8 (73.4, 106.5)	0.15 (0.14, 0.16)	241.9 (127.2, 452.4)	0.21 (0.16, 0.26)	155.2 (135.1, 179.4)	0.19 (0.18, 0.20)	1291.9 (1067.5, 1546.1)	0.41 (0.39, 0.42)
10:00	8.8 (6.9, 11.2)	0.09 (0.09, 0.10)	99.3 (83.9, 117.7)	0.14 (0.13, 0.15)	124.4 (88.2, 180.0)	0.16 (0.14, 0.19)	121.7 (100.7, 144.9)	0.17 (0.15, 0.18)	282.8 (133.2, 587.7)	0.22 (0.17, 0.27)	160.8 (140.8, 183.2)	0.19 (0.18, 0.20)	1734.1 (1453.6, 2084.7)	0.42 (0.40, 0.44)
11:00	8.8 (6.9, 11.2)	0.09 (0.09, 0.10)	102.1 (86.2, 120.2)	0.14 (0.14, 0.15)	118.2 (85.1, 163.7)	0.15 (0.13, 0.17)	144.6 (119.6, 172.5)	0.17 (0.16, 0.19)	444.4 (251.1, 769.8)	0.22 (0.18, 0.27)	193.8 (168.9, 221.7)	0.20 (0.19, 0.21)	1585.0 (1317.2, 1899.3)	0.42 (0.40, 0.44)
12:00	8.8 (6.9, 11.2)	0.09 (0.09, 0.10)	107.6 (91.1, 127.5)	0.14 (0.13, 0.15)	101.7 (77.0, 138.1)	0.14 (0.12, 0.16)	149.0 (122.7, 179.6)	0.18 (0.17, 0.19)	278.1 (194.7, 409.2)	0.22 (0.19, 0.25)	199.3 (174.4, 228.5)	0.20 (0.19, 0.22)	1392.3 (1165.9, 1649.2)	0.41 (0.39, 0.42)
13:00	8.8 (6.9, 11.2)	0.09 (0.09, 0.10)	99.5 (83.6, 119.8)	0.14 (0.13, 0.15)	163.0 (117.5, 222.0)	0.16 (0.14, 0.18)	148.0 (123.8, 177.9)	0.18 (0.17, 0.19)	360.9 (219.3, 585.9)	0.24 (0.20, 0.27)	191.1 (167.6, 218.3)	0.21 (0.20, 0.22)	1362.1 (1147.0, 1614.8)	0.40 (0.38, 0.42)
14:00	8.9 (6.9, 11.2)	0.09 (0.09, 0.10)	91.1 (77.4, 107.8)	0.14 (0.13, 0.15)	132.7 (93.3, 191.2)	0.16 (0.14, 0.18)	131.5 (108.3, 156.8)	0.17 (0.15, 0.18)	718.0 (386.3, 1354.8)	0.31 (0.26, 0.35)	185.8 (162.5, 213.9)	0.20 (0.19, 0.22)	1314.9 (1106.7, 1566.6)	0.40 (0.38, 0.42)
15:00	8.9 (6.9, 11.2)	0.09 (0.09, 0.10)	72.9 (61.9, 85.8)	0.12 (0.11, 0.13)	124.4 (92.0, 170.1)	0.16 (0.14, 0.18)	120.1 (100.0, 141.9)	0.16 (0.15, 0.17)	178.0 (86.4, 361.9)	0.22 (0.16, 0.27)	174.5 (152.5, 199.0)	0.2 (0.19, 0.21)	1075.0 (906.9, 1273.7)	0.39 (0.37, 0.41)
16:00	8.9 (6.9, 11.2)	0.09 (0.09, 0.10)	54.8 (47.3, 64.1)	0.1 (0.09, 0.11)	74.0 (59.2, 93.7)	0.12 (0.10, 0.13)	92.8 (76.9, 110.4)	0.15 (0.14, 0.16)	112.3 (48.7, 257.3)	0.19 (0.12, 0.25)	197.9 (171.3, 231.5)	0.21 (0.20, 0.22)	753.0 (636.8, 893.7)	0.36 (0.35, 0.38)
17:00	18.8 (13.3, 25.6)	0.06 (0.06, 0.07)	39.8 (34.8, 45.9)	0.08 (0.08, 0.09)	60.6 (49.8, 72.0)	0.10 (0.09, 0.11)	80.2 (66.3, 96.3)	0.14 (0.13, 0.15)	42.1 (25.5, 66.9)	0.12 (0.08, 0.16)	138.3 (117.4, 160.3)	0.18 (0.17, 0.19)	457.3 (386.4, 538.7)	0.32 (0.31, 0.34)
18:00	11.6 (9.0, 14.9)	0.04 (0.03, 0.04)	32.2 (28.3, 36.5)	0.07 (0.06, 0.07)	44.0 (37.5, 52.1)	0.08 (0.07, 0.09)	56.6 (45.8, 68.9)	0.12 (0.10, 0.13)	30.8 (22.0, 44.7)	0.09 (0.06, 0.12)	77.3 (64.1, 91.9)	0.14 (0.13, 0.16)	287.9 (239.0, 341.9)	0.29 (0.28, 0.31)

19:00	11.6 (9.1, 14.6)	0.04 (0.04, 0.05)	22.9 (20.5, 25.9)	0.05 (0.05, 0.06)	30.1 (25.5, 35.8)	0.06 (0.05, 0.08)	29.2 (23.6, 35.9)	0.07 (0.06, 0.09)	21.6 (14.7, 31.5)	0.07 (0.05, 0.10)	55.9 (46.6, 67.1)	0.12 (0.10, 0.14)	130.9 (104.5, 163.2)	0.23 (0.21, 0.25)
20:00	5.9 (4.8, 7.1)	0.03 (0.02, 0.03)	16.5 (14.8, 18.4)	0.04 (0.04, 0.05)	23.1 (18.9, 28.7)	0.05 (0.03, 0.06)	14.6 (11.4, 18.8)	0.04 (0.02, 0.06)	11.7 (7.3, 18.2)	0.04 (0.00, 0.07)	27.7 (22.3, 33.9)	0.08 (0.06, 0.10)	39.8 (28.5, 53.5)	0.11 (0.08, 0.14)
21:00	2.8 (2.3, 3.3)	0.01 (0.01, 0.02)	14.8 (13.3, 16.5)	0.04 (0.03, 0.04)	18.2 (14.13, 23.6)	0.04 (0.02, 0.06)	11.2 (8.2, 15.1)	0.04 (0.02, 0.06)	15.3 (8.7, 28.1)	0.04 (-0.02, 0.08)	14.7 (10.8, 19.4)	0.05 (0.03, 0.08)	17.4 (9.8, 30.4)	0.05 (-0.01, 0.11)
22:00	2 (1.7, 2.3)	0.01 (0.01, 0.01)	13.7 (12.2, 15.6)	0.04 (0.03, 0.04)	19.3 (13.2, 27.8)	0.05 (0.02, 0.07)	11.2 (7.8, 15.6)	0.04 (0.01, 0.06)	7.3 (3.4, 15.7)	0.03 (-0.02, 0.08)	6.5 (5.4, 7.9)	0.07 (0.06, 0.09)	3.5 (1.8, 7.0)	0.03 (-0.04, 0.10)
23:00	1.2 (1.0, 1.3)	0.01 (0.00, 0.01)	9.6 (8.4, 11.2)	0.03 (0.02, 0.04)	18.5 (12.7, 26.7)	0.04 (0.01, 0.07)	5.6 (3.9, 7.9)	0.02 (0.00, 0.05)	4.5 (1.6, 12.3)	0.03 (-0.05, 0.11)	6.5 (5.4, 7.9)	0.07 (0.06, 0.09)	3.5 (1.8, 7.0)	0.03 (-0.04, 0.10)

*Some hours of the day categories were collapased and estimated together due to limited data.

Supplementary Table S4. Estimated illuminance (lux) and circadian stimulus (CS) by time of day and location with boot strapped 95% confidence limits for 5 light environments.

	Darkness		Household light[a]		Indoors Natural light only		Non-Residential light[b]		Outdoors in Daylight	
Time[c]	lux	CS	lux	CS	lux	CS	lux	CS	lux	CS
0:00	1.0 (0.9, 1.1)	0.00 (0.00, 0.01)	9.4 (7.8, 11.5)	0.03 (0.02, 0.04)	5.6 (3.9, 7.9)	0.02 (0.00, 0.05)	6.2 (5.1, 7.5)	0.07 (0.05, 0.08)	3.5 (1.8, 7.0)	0.03 (-0.04, 0.1)
1:00	0.9 (0.8, 1.0)	0.00 (0.00, 0.00)	8.2 (6.4, 10.2)	0.03 (0.02, 0.05)	5.6 (3.9, 7.9)	0.02 (0.00, 0.05)	6.2 (5.1, 7.5)	0.07 (0.05, 0.08)	3.5 (1.8, 7.0)	0.03 (-0.04, 0.1)
2:00	0.9 (0.8, 1.0)	0.00 (0.00, 0.00)	6.4 (5.0, 8.5)	0.03 (0.01, 0.04)	5.6 (3.9, 7.9)	0.02 (0.00, 0.05)	6.2 (5.1, 7.5)	0.07 (0.05, 0.08)	3.5 (1.8, 7.0)	0.03 (-0.04, 0.1)
3:00	0.9 (0.8, 1.0)	0.00 (0.00, 0.00)	8.4 (6.4, 10.9)	0.03 (0.02, 0.05)	5.6 (3.9, 7.9)	0.02 (0.00, 0.05)	6.2 (5.1, 7.5)	0.07 (0.05, 0.08)	3.5 (1.8, 7.0)	0.03 (-0.04, 0.1)
4:00	1.0 (0.9, 1.2)	0.01 (0.00, 0.01)	13.0 (10.5, 16.1)	0.05 (0.04, 0.07)	5.6 (3.9, 7.9)	0.02 (0.00, 0.05)	6.2 (5.1, 7.5)	0.07 (0.05, 0.08)	3.5 (1.8, 7.0)	0.03 (-0.04, 0.1)
5:00	1.3 (1.2, 1.5)	0.01 (0.01, 0.01)	19.5 (16.7, 22.3)	0.06 (0.05, 0.07)	4.9 (3.4, 7.0)	0.03 (0.00, 0.05)	16.7 (12.3, 22.7)	0.09 (0.06, 0.12)	35.1 (16.6, 70.0)	0.10 (0.02, 0.18)
6:00	2.1 (1.8, 2.4)	0.02 (0.02, 0.02)	34.5 (30.3, 39.0)	0.08 (0.08, 0.09)	7.0 (5.7, 8.6)	0.04 (0.03, 0.06)	68.3 (53.4, 86.2)	0.13 (0.11, 0.15)	92.5 (68.7, 122.4)	0.20 (0.17, 0.23)
7:00	3.8 (3.2, 4.6)	0.03 (0.03, 0.04)	47.9 (42.3, 53.7)	0.10 (0.10, 0.11)	15.2 (12.7, 18.2)	0.07 (0.06, 0.09)	123.5 (105.0, 146.7)	0.17 (0.15, 0.18)	307.5 (254.0, 370.2)	0.3 (0.28, 0.32)
8:00	4.4 (3.4, 5.7)	0.06 (0.05, 0.07)	76.6 (66.9, 87.2)	0.12 (0.12, 0.13)	44.8 (37.4, 53.1)	0.12 (0.10, 0.13)	151.5 (131.9, 174.2)	0.19 (0.18, 0.20)	713.5 (596.3, 848.3)	0.36 (0.34, 0.38)
9:00	2.6 (1.8, 3.6)	0.02 (0.01, 0.03)	89.1 (76.2, 103.2)	0.13 (0.13, 0.14)	88.8 (73.4, 106.5)	0.15 (0.14, 0.16)	156.4 (136.0, 177.7)	0.19 (0.18, 0.20)	1291.9 (1067.5, 1546.1)	0.41 (0.39, 0.42)
10:00	8.8 (6.9, 11.2)	0.09 (0.09, 0.10)	102.9 (88.0, 119.3)	0.14 (0.14, 0.15)	121.7 (100.7, 144.9)	0.17 (0.15, 0.18)	160.8 (141.1, 184.6)	0.19 (0.18, 0.20)	1734.1 (1453.6, 2084.7)	0.42 (0.40, 0.44)
11:00	8.8 (6.9, 11.2)	0.09 (0.09, 0.10)	105.8 (90.1, 124.4)	0.15 (0.14, 0.15)	144.6 (119.6, 172.5)	0.17 (0.16, 0.19)	196.7 (172.0, 226.0)	0.2 (0.19, 0.21)	1585.0 (1317.2, 1899.3)	0.42 (0.40, 0.44)
12:00	8.8 (6.9, 11.2)	0.09 (0.09, 0.10)	107.0 (91.5, 126.8)	0.14 (0.14, 0.15)	149.0 (122.7, 179.6)	0.18 (0.17, 0.19)	200.7 (174.8, 230.7)	0.21 (0.19, 0.22)	1392.3 (1165.9, 1649.2)	0.41 (0.39, 0.42)
13:00	8.8 (6.9, 11.2)	0.09 (0.09, 0.10)	109.4 (93.3, 127.5)	0.14 (0.14, 0.15)	148.0 (123.8, 177.9)	0.18 (0.17, 0.19)	193.4 (169.0, 220.0)	0.21 (0.19, 0.22)	1362.1 (1147.0, 1614.8)	0.40 (0.38, 0.42)
14:00	8.9 (6.9, 11.2)	0.09 (0.09, 0.10)	97.3 (83.9, 112.5)	0.14 (0.13, 0.15)	131.5 (108.3, 156.8)	0.17 (0.15, 0.18)	188.9 (164.4, 216.9)	0.21 (0.19, 0.22)	1314.9 (1106.7, 1566.6)	0.40 (0.38, 0.42)
15:00	8.9 (6.9, 11.2)	0.09 (0.09, 0.10)	80.7 (69.1, 94.3)	0.13 (0.12, 0.14)	120.1 (100.0, 141.9)	0.16 (0.15, 0.17)	173.3 (151.7, 198.7)	0.20 (0.19, 0.21)	1075.0 (906.9, 1273.7)	0.39 (0.37, 0.41)
16:00	8.9 (6.9, 11.2)	0.09 (0.09, 0.10)	58.7 (51.5, 67.0)	0.11 (0.10, 0.11)	92.8 (76.9, 110.4)	0.15 (0.14, 0.16)	194.7 (169.5, 226.0)	0.21 (0.20, 0.22)	753.0 (636.8, 893.7)	0.36 (0.35, 0.38)
17:00	18.8 (13.3, 25.6)	0.06 (0.06, 0.07)	45.4 (40.3, 51.2)	0.09 (0.08, 0.10)	80.2 (66.3, 96.3)	0.14 (0.13, 0.15)	124.2 (106.4, 146.4)	0.18 (0.16, 0.19)	457.3 (386.4, 538.7)	0.32 (0.31, 0.34)

18:00	11.6 (9.0, 14.9)	0.04 (0.03, 0.04)	35.1 (31.2, 39.4)	0.07 (0.07, 0.08)	56.6 (45.8, 68.9)	0.12 (0.10, 0.13)	60.6 (51.7, 71.7)	0.13 (0.11, 0.14)	287.9 (239.0, 341.9)	0.29 (0.28, 0.31)
19:00	11.6 (9.1, 14.6)	0.04 (0.04, 0.05)	24.0 (21.6, 26.8)	0.06 (0.05, 0.06)	29.2 (23.6, 35.9)	0.07 (0.06, 0.09)	44.2 (37.4, 52.4)	0.11 (0.09, 0.12)	130.9 (104.5, 163.2)	0.23 (0.21, 0.25)
20:00	5.9 (4.8, 7.1)	0.03 (0.02, 0.03)	16.9 (15.2, 18.8)	0.04 (0.04, 0.05)	14.6 (11.4, 18.8)	0.04 (0.02, 0.06)	21.5 (17.5, 25.9)	0.06 (0.05, 0.08)	39.8 (28.5, 53.5)	0.11 (0.08, 0.14)
21:00	2.8 (2.3, 3.3)	0.01 (0.01, 0.02)	14.7 (13.2, 16.3)	0.04 (0.03, 0.04)	11.2 (8.2, 15.1)	0.04 (0.02, 0.06)	14.5 (11.1, 18.7)	0.05 (0.03, 0.07)	17.4 (9.8, 30.4)	0.05 (-0.01, 0.11)
22:00	2 (1.7, 2.3)	0.01 (0.01, 0.01)	13.6 (12.1, 15.4)	0.04 (0.03, 0.04)	11.2 (7.8, 15.6)	0.04 (0.01, 0.06)	6.2 (5.1, 7.5)	0.07 (0.05, 0.08)	3.5 (1.8, 7.0)	0.03 (-0.04, 0.10)
23:00	1.2 (1.0, 1.3)	0.01 (0.00, 0.01)	9.9 (8.6, 11.2)	0.03 (0.03, 0.04)	5.6 (3.9, 7.9)	0.02 (0.00, 0.05)	6.2 (5.1, 7.5)	0.07 (0.05, 0.08)	3.5 (1.8, 7.0)	0.03 (-0.04, 0.10)

[a] Includes Kitchen light with all other Household light
[b] Includes Restaurant/Hotel light with Non-Residential light
[c] Some hours of the day categories were collapsed and estimated together due to limited data.

Supplementary Figure S1. Percent agreement between the 4 weekly-diaries and the annual survey in the same year by time of day for all days (red triangles with solid lines), workdays (blue squares with dash-dot lines), and non-workdays (green circles with dotted lines).

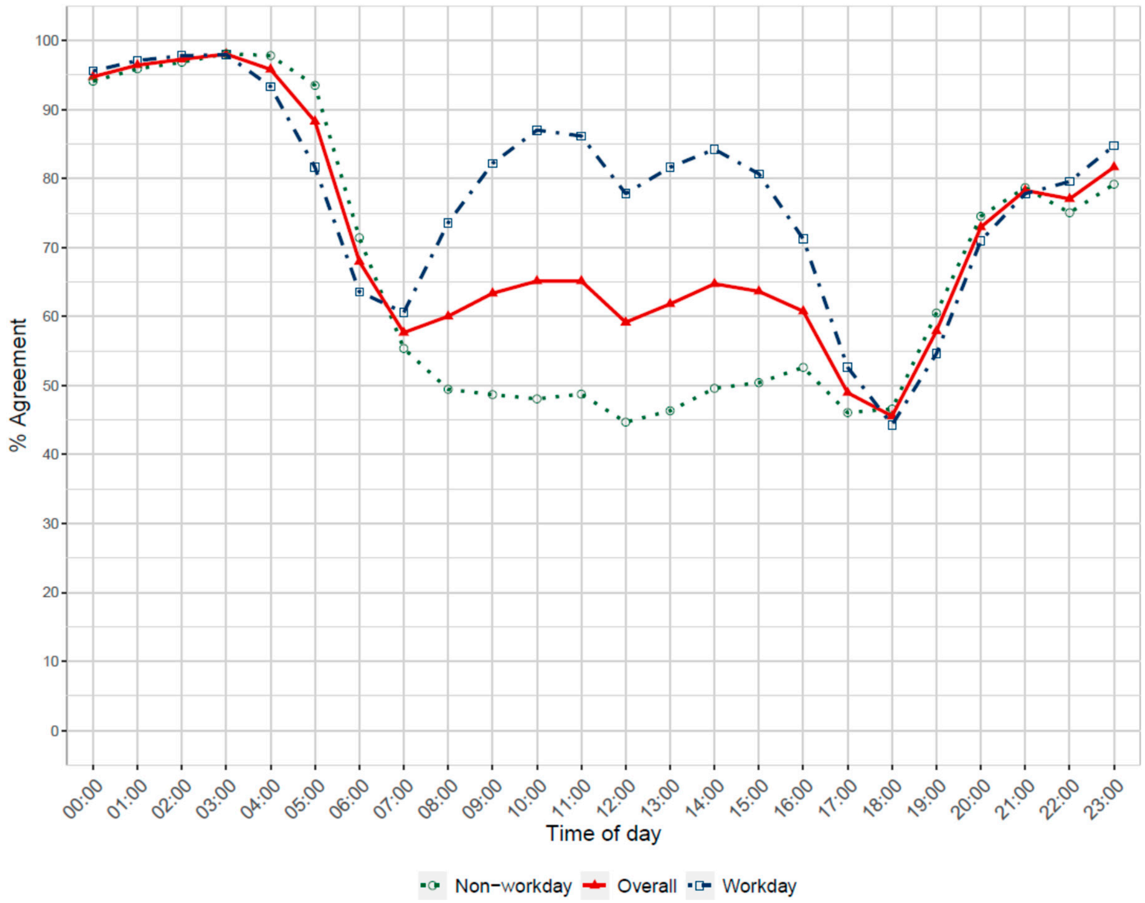

Supplement: Supplementary file 1 [file ijerph-20-03658-s001.zip › ijerph-2147925-supplementary.pdf]
